# Evidence of aberrant anti-epstein-barr virus antibody response, though no viral reactivation, in people with post-stroke fatigue

**DOI:** 10.1186/s12950-024-00402-0

**Published:** 2024-08-12

**Authors:** Isobel C. Mouat, Li Zhu, Alperen Aslan, Barry W. McColl, Stuart M. Allan, Craig J. Smith, Marion S. Buckwalter, Laura McCulloch

**Affiliations:** 1 Centre for Inflammation Research, Institute for Regeneration and Repair South, University of Edinburgh, Edinburgh, UK; 2 Department of Neurology and Neurological Sciences, Stanford University School of Medicine, Stanford, USA; 3 UK Dementia Research Institute, Centre for Discovery Brain Sciences, The University of Edinburgh, Edinburgh, UK; 4 Geoffrey Jefferson Brain Research Centre, School of Biological Sciences, Faculty of Biology, Medicine and Health, The University of Manchester, Manchester, UK; 5 Division of Neuroscience, The University of Manchester, Manchester, UK; 6 Lydia Becker Institute of Immunology and Inflammation, Faculty of Biology, Medicine and Health, Manchester Academic Health Science Centre, The University of Manchester, Manchester, UK; 7 Division of Cardiovascular Sciences, The University of Manchester, Manchester, UK; 8 Geoffrey Jefferson Brain Research Centre, Manchester Academic Health Science Centre, Northern Care Alliance NHS Foundation Trust, University of Manchester, Manchester, UK; 9 Wu Tsai Neurosciences Institute, Stanford University, Stanford, CA USA; 10 Department of Neurosurgery, Stanford University School of Medicine, Stanford, CA USA

**Keywords:** Ischaemic stroke, Fatigue, Epstein-Barr virus

## Abstract

**Background:**

Fatigue is a common complication of stroke that has a significant impact on quality of life. The biological mechanisms that underly post-stroke fatigue are currently unclear, however, reactivation of latent viruses and their impact on systemic immune function have been increasingly reported in other conditions where fatigue is a predominant symptom. Epstein-Barr virus (EBV) in particular has been associated with fatigue, including in long-COVID and myalgic encephalomyelitis/chronic fatigue syndrome, but has not yet been explored within the context of stroke.

**Aims:**

We performed an exploratory analysis to determine if there is evidence of a relationship between EBV reactivation and post-stroke fatigue.

**Methods:**

In a chronic ischemic stroke cohort (> 5 months post-stroke), we assayed circulating EBV by qPCR and measured the titres of anti-EBV antibodies by ELISA in patients with high fatigue (FACIT-F < 40) and low fatigue (FACIT-F > 41). Statistical analysis between two-groups were performed by t-test when normally distributed according to the Shapiro-Wilk test, by Mann-Whitney test when the data was not normally distributed, and by Fisher’s exact test for categorical data.

**Results:**

We observed a similar incidence of viral reactivation between people with low versus high levels of post-stroke fatigue (5 of 22 participants (24%) versus 6 of 22 participants (27%)). Although the amount of circulating EBV was similar, we observed an altered circulating anti-EBV antibody profile in participants with high fatigue, with reduced IgM against the Viral Capsid Antigen (2.244 ± 0.926 vs. 3.334 ± 2.68; *P* = 0.031). Total IgM levels were not different between groups indicating this effect was specific to anti-EBV antibodies (3.23 × 10^5^ ± 4.44 × 10^4^ high fatigue versus 4.60 × 10^5^ ± 9.28 × 10^4^ low fatigue; *P* = 0.288).

**Conclusions:**

These data indicate that EBV is not more prone to reactivation during chronic stroke recovery in those with post-stroke fatigue. However, the dysregulated antibody response to EBV may be suggestive of viral reactivation at an earlier stage after stroke.

**Supplementary Information:**

The online version contains supplementary material available at 10.1186/s12950-024-00402-0.

## Introduction

Post-stroke fatigue is a major clinical concern that is experienced by around half of stroke survivors [1]. Fatigue has a major impact on quality of life and has been identified by stroke survivors, carers and health care professionals as a high priority for future research [2]. Post-stroke fatigue is defined as “a feeling of exhaustion, weariness or lack of energy that can be overwhelming, and which can involve physical, emotional, cognitive and perceptual contributors, which is not relieved by rest and affects a person’s daily life” [3]. Multiple factors are hypothesised to contribute to risk of post-stroke fatigue, including biological sex, white matter abnormalities, stroke severity, and depression [4]. Despite its clinical importance, there are currently no effective treatments, though there are recent promising reports on the efficacy of Modafinil [5]. Identifying biological mechanisms driving post-stroke fatigue has been recommended as a research priority, with dysregulation of the immune system and inflammation highlighted as the top area for further investigation [3].

With the recent emergence of long-COVID, there has been an upsurge of interest in understanding common factors that drive fatigue in different clinical conditions. Reactivation of Epstein-Barr virus (EBV) and its association with a dysregulated immune system has been increasingly associated with fatigue, including in long-COVID [6] and myalgic encephalomyelitis/chronic fatigue syndrome (ME/CFS) [7]. EBV is a ubiquitous gamma-herpesvirus virus that infects > 90% of all people [8]. EBV lies dormant for the majority of life, and constant host immune surveillance largely maintains the virus in a latent state. There can be however periodic reactivation, particularly during periods of immunosuppression [9]. EBV reactivation has been observed in an array of conditions where the immune system is compromised, including haematopoietic and solid organ transplantation [10, 11], HIV infection [12, 13], and stress [14]. While it is still unknown how EBV is linked to the development of fatigue, it is thought that genetic susceptibility [15] and viral-induced chronic inflammation [16] may both have a role.

Suppression of some aspects of systemic immune function is a common feature of stroke and is associated with infection vulnerability and worse outcomes [17–20]. Therefore, post-stroke immune suppression has potential to create an immune environment permissible to reactivation of EBV. This has been shown for other latent viruses as in the five years following stroke there is an increased risk of herpes zoster reactivation [21]. Animal models indicate risk of both HIV and herpes simplex virus 1 (HSV-1) reactivation following stroke [22, 23]. Here, we focus on EBV due to its high seroprevalence. In this study we aimed to investigate the frequency of circulating EBV and the immunoglobulin response to EBV in people recovering from stroke with high versus low levels of reported fatigue.

## Methods

### Study design and participants

The StrokeCog study is a longitudinal cohort study aiming to investigate the post-stroke immune response and cognitive functioning over the course of stroke recovery [24]. Participants included in the parent study had ischemic strokes that were confirmed by MRI or CT scan, were over the age of 25, were able to return for annual follow-up visits, and had fluency in English. Exclusion criteria included preceding cognitive impairment or dysphasia that precluded ability to complete the neurological assessments, life expectancy of under one year, or pre-existing conditions that would impact the assessment of neurological or cognitive outcomes. The parent study was approved by the Stanford University Institutional Review Board. We designed this as a nested case-control (fatigue vs. no fatigue) cohort within the StrokeCog cohort. Cohort characteristics are further discussed in the results section. The study was conducted at Stanford School of Medicine (Stanford, CA, USA) and was approved by Stanford’s Institutional Review Board, eprotocol number 42,089. It is in compliance with Good Clinical Practices (ICH/GCP) as consistent with US Food and Drug Administration Code of Federal Regulations (21 CFR 50 and 56) and DHHS Regulations (45 CFR Part 46). Written informed consent was obtained from participants in person.

### Clinical data

A neurological evaluation was performed on each participant comprised of various neuropsychological assessments [24]. For the purpose of this study, we are particularly interested in fatigue, mood, and cognition. Fatigue was measured using the Functional Assessment of Chronic Illness Therapy – Fatigue Scale (FACIT-F) [25], a questionnaire tool that assesses patient-reported fatigue and impact on daily living. The measure is made up of 13 questions, each requiring an answer on a 5-point Likert-type scale, with the summation of responses producing a single value. Additionally, the Stroke Impact Scale (SIS) version 3 was administered, a well-established patient-based, self-report questionnaire [26]. Various domains are measured, and for the purpose of this study we examined the SIS3 domain, which is a measure of mood/emotion. Finally, to measure cognition, the Montreal Cognitive Assessment (MoCA) was utilized [27].

### Sample collection and processing

Blood was collected the same time as neurological assessment. For the isolation of plasma and peripheral blood mononuclear cells (PBMCs), blood was collected into sterile BD Vacutainer tubes containing 15% K3 ethylenediaminetetraacetic acid (EDTA) solution (Thermo Fisher Scientific), placed on ice, and centrifuged at 2,000 g for 10 min at 4 °C. Plasma supernatant was isolated, aliquoted, and stored at -80 °C prior to use. The remaining sample was further processed with a 15mL Ficoll density gradient in a SepMate Tube-50, cells were washed with PBS with 2% fetal bovine serum (FBS), counted on a haemocytometer, and frozen in a Mr. Frosty (Thermo Fisher) at -80 °C for 24 h before being transferred to liquid nitrogen for storage. Genomic DNA was extracted from 2 × 10^6^ PBMCs with the Qiagen QIAamp DNA Blood Mini Kit (cat no 51104).

### Detection of antibodies

Enzyme-linked immunosorbent assay (ELISA) was used to measure antibody titres in plasma including those against EBV antigens and C-reactive protein (CRP) and total IgM and IgG. Commercial ELISA kits were used to measure anti-viral capsid antigen (VCA) IgM (Abcam, ab108732), anti-VCA IgG (Abcam, ab108730), anti-Epstein-Barr nuclear antigen 1 (EBNA1) IgG (Abcam, ab108731), IgM (Abcam, ab214568), IgG (Abcam, ab195215), and CRP (Abcam, ab260058), and completed according to manufacturer’s instructions. Plasma samples were diluted to 1:100 concentration for the anti-VCA IgM and anti-EBNA1 IgG, 1:2000 for the anti-VCA IgG and CRP and 1:100,000 for the IgM and total IgG. Due to the capacity of the plates, two participants from each group were randomly removed from each analysis. Samples were run in duplicate and each plate was run with positive, negative, and cut-off controls (anti-VCA and anti-EBNA1), or a standard. Absorbance was measured at 450 nm using a SpectraMax 340PC384 (Molecular Devices). Interpretation of results was done in accordance with manufacturer’s instructions. For anti-EBV titres, samples were considered to give a positive result if more than 10% above the cut-off control.

### EBV viraemia

Circulating EBV was measured by qPCR with gDNA isolated from peripheral leukocytes. The Norgen EBV TaqMan PCR kit (TM41050) was used and duplicate samples were run on a BioRad CFX96 Real-Time PCR Detection System (BioRad). The assay was completed and results were analysed in accordance with manufacturer’s instructions. All samples displayed amplification of the internal PCR validation control.

### Statistical analyses

Analysis and presentation of data was performed with GraphPad Prism software version 9.5.1 (GraphPad Software Inc.). Primary analysis compared the frequency of circulating EBV and EBV viral load between fatigued and non-fatigued stroke groups. Exploratory secondary analyses were the comparison of antibodies titres specific to EBV antigens load between fatigued and non-fatigued stroke groups. Statistical analysis between two-groups were performed by t-test when normally distributed according to the Shapiro-Wilk test, by Mann-Whitney test when the data was not normally distributed, and by Fisher’s exact test for categorical data. Correlation analyses were performed by simple linear regression on log-transformed data, with best-fit line and 95% confidence bands plotted. *P* < 0.05 was considered statistically significant.

## Results

### Cohort selection

**Fig. 1:**
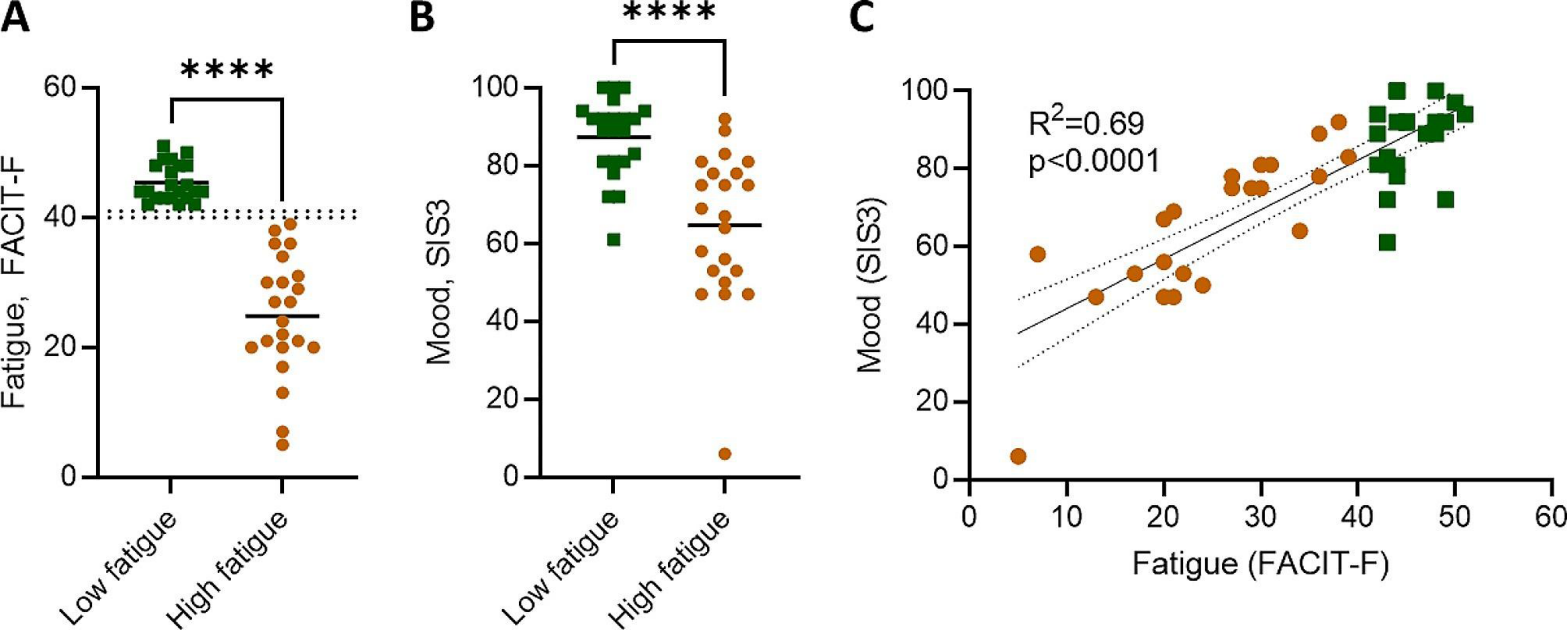
Fatigue and mood of cohort. (**A**) Fatigue, measured by the Functional Assessment of Chronic Illness Therapy – Fatigue (FACIT-F) tool, with threshold for high versus low fatigue demarcated by dotted lines (**B**) mood, as measured by the Stroke Impact Scale (SIS), and correlation between fatigue (FACIT-F) and mood (SIS3). Each data point represents an individual participant, *n* = 22 per group. Data show individual participants plus mean; analysed by (**A**, **B**) Mann-Whitney test and (**C**) linear regression, with best-fit line and 95% confidence band plotted; ****P < 0.0001

A total of 44 participants from the StrokeCog cohort were included in this exploratory study based on their experience of fatigue. Patients completed a fatigue questionnaire alongside a battery of neurological tests at a minimum of five months post stroke onset and a concurrent blood sample was taken. To identify participants for this study we selected the 30 individuals with the highest FACIT-F scores and 30 participants with the lowest FACIT-F scores, and of those identified 22 participants in each group that matched with regards to age, biological sex, infarct volume, initial stroke severity (National Institutes of Health Stroke Scale, NIHSS), and time from stroke to sample collection (Table 1). The resulting cohort comprised of a high fatigue (FACIT-F < 40) and low fatigue (FACIT-F > 41) group (Fig. 1A), with the dichotomisation aligning with previously published values for levels of post-stroke fatigue [28]. The higher fatigue group had lower Stroke Impact Scale domain 3 (SIS3) scores, indicating worse mood, and there was association between fatigue (FACIT-F) and mood (SIS3) (Fig. 1B, C). The cohort was comprised of individuals with relatively low severity strokes, with a median NIHSS of 4 and 5 for the low and high fatigue groups, respectively, and a median infarct volume of 7.2mL (low fatigue) and 5.5mL (high fatigue, Table 1). The low and high fatigue groups had similar, and not significantly different, prevalences of risk factors, including hypertension and previous stroke, as well as medication usage. However, there was a small, but not significant, increase in duration from index stroke in the high fatigue group (Table 1).

**Table 1:** Clinical cohort

	High fatigue	Low fatigue
**Age at Baseline**, median (IQR)	65 (56–71)	66 (58–71)
**Sex**		
Male, n (%)	14 (64)	14 (64)
Female, n (%)	8 [36]	8 [36]
**Presentation**		
Right hemisphere, n (%)	13 (59%)	10 (45%)
Left hemisphere, n (%)	9 (41%)	10 (45%)
Bilateral, n (%)	0 (0%)	2 (9%)
NIHSS, median (IQR)	5 [2–11]	4 [2–13]
Infarct volume (mL), median (IQR)	5.5 (0.46–39.08)	7.2 (1.98–42.33)
Days from Stroke to Baseline, median (IQR)	316 (235–1062)	256 (206–1236)
IV thrombolysis, n (%)	4 (20%)	8 (38%)
IA thrombectomy, n (%)	4 (20%)	6 (29%)
**Risk Factors**		
Hypertension, n (%)	15 (75%)	9 (43%)
Previous stroke, n (%)	4 (24%)	2 (12%)
Atrial fibrillation, n (%)	3 (17%)	5 (24%)
Hyperlipidaemia, n (%)	9 (53%)	6 (29%)
Other cardiovascular diseases, n (%)	2 (12%)	3 (17%)
Heart valve repair/replacement, n (%)	1 (5%)	2 (9%)
Angina, n (%)	0 (0%)	0 (%)
Congestive heart failure, n (%)	1 (5%)	1 (5%)
Pacemaker and/or defibrillator, n (%)	0 (0%)	2 (9%)
Coronary angioplasty, n (%)	3 (15%)	4 (18%)
**Medications**		
Antiplatelets, n (%)	6 (27%)	5 (23%)
Anticoagulants, n (%)	1 (5%)	2 (9%)
Statin, n (%)	18 (82%)	16 (73%)
Beta-blocker, n (%)	6 (27%)	6 (27%)
ACE-inhibitor, n (%)	4 (18%)	4 (18%)
Angiotensin II receptor blocker, n (%)	6 (27%)	4 (18%)

Median and interquartile range (IQR), analysed by Mann-Whitney test for nonparametric data and Fisher’s exact test for categorical data. NIHSS: National Institutes of Health Stroke Scale; IV: intravenous; IA: intra-arterial; ACE: Angiotensin Converting Enzyme

### Frequency of circulating virus is not different between participants with high and low post-stroke fatigue

**Fig. 2:**
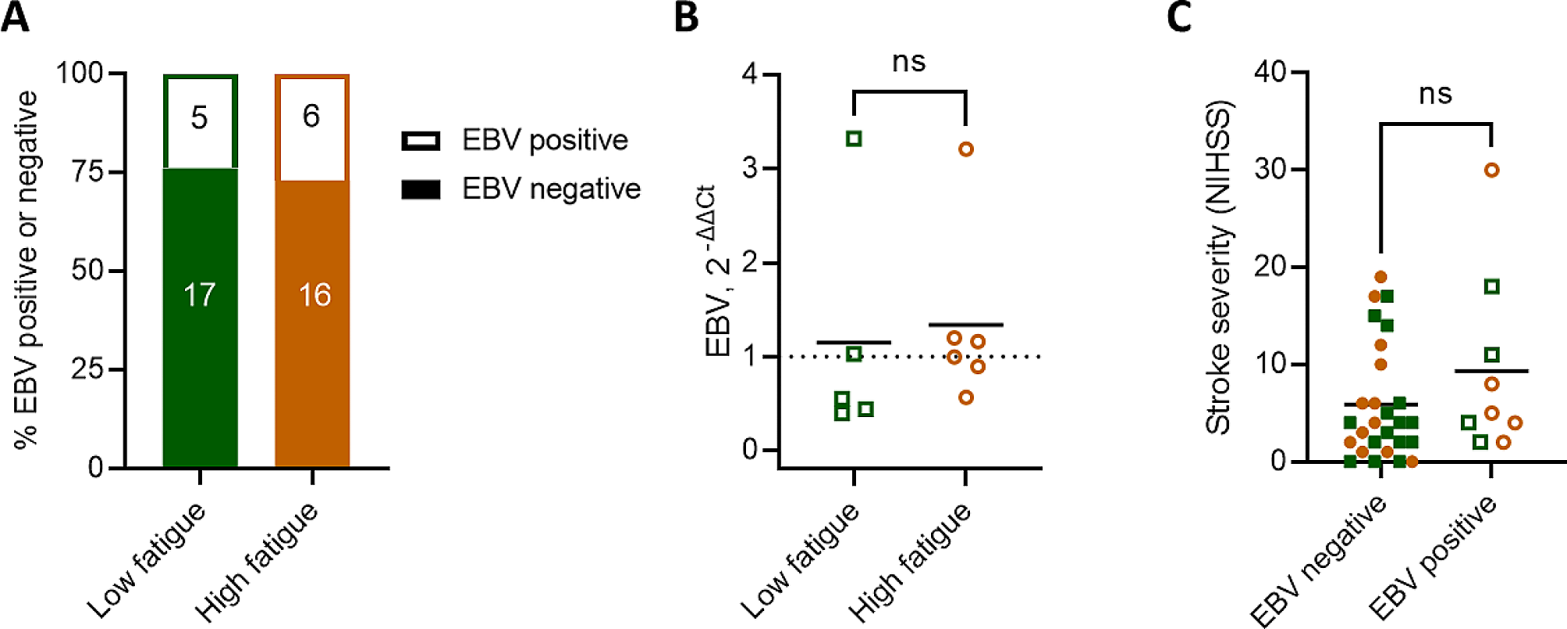
Similar frequencies of EBV viraemia in people with and without post-stroke fatigue. (**A**) Frequency of circulating EBV measured by qPCR. (**B**) EBV load assessed in the subset of participants positive for EBV quantified with the delta-delta Ct method, using the housekeeping gene provided by the Norgen EBV PCR kit. (**C**) Stroke severity, measured by NIHSS, between participants with and without detectable circulating EBV by qPCR. Low fatigue participants are plotted as green squares and high fatigue as orange circles. Open shapes indicate participants with EBV viremia, as measured by qPCR. Data show individual participants plus mean, (**A**) *n* = 22, (**B**) *n* = 5–6, (**C**) *n* = 9–27 per group; (**B**) unpaired t-test; (**C**) Mann-Whitney test

EBV is expected to be detectable in circulation during primary infection and reactivation events, though not during latency when the virus lays dormant in lymphoid tissues. EBV DNA was detected in 5 of 22 participants (24%) with low fatigue and 6 of 22 participants (27%) with high fatigue (Fig. 2A). Of the individuals positive for EBV, no difference was observed in the viral load between those with low and high fatigue (Fig. 2B). Between participants with and without detectable circulating EBV, there was no difference in the initial stroke severity, measured by NIHSS (Fig. 2C).

### EBV seroresponse in people with post-stroke fatigue

To investigate the antibody response to EBV, we measured circulating antibodies against the EBV lytic antigen VCA and latency antigen EBNA1. All participants were seropositive for EBV, as measured by the presence of at least one IgG antibody class against EBNA1, IgG against VCA, or IgM against VCA. No recent primary infections were detected, defined as a positive IgM anti-VCA and negative IgG anti-EBNA1 (data not shown). Therefore, all patients showing positive viremia by qPCR were experiencing reactivation of previously latent infection.

**Fig. 3:**
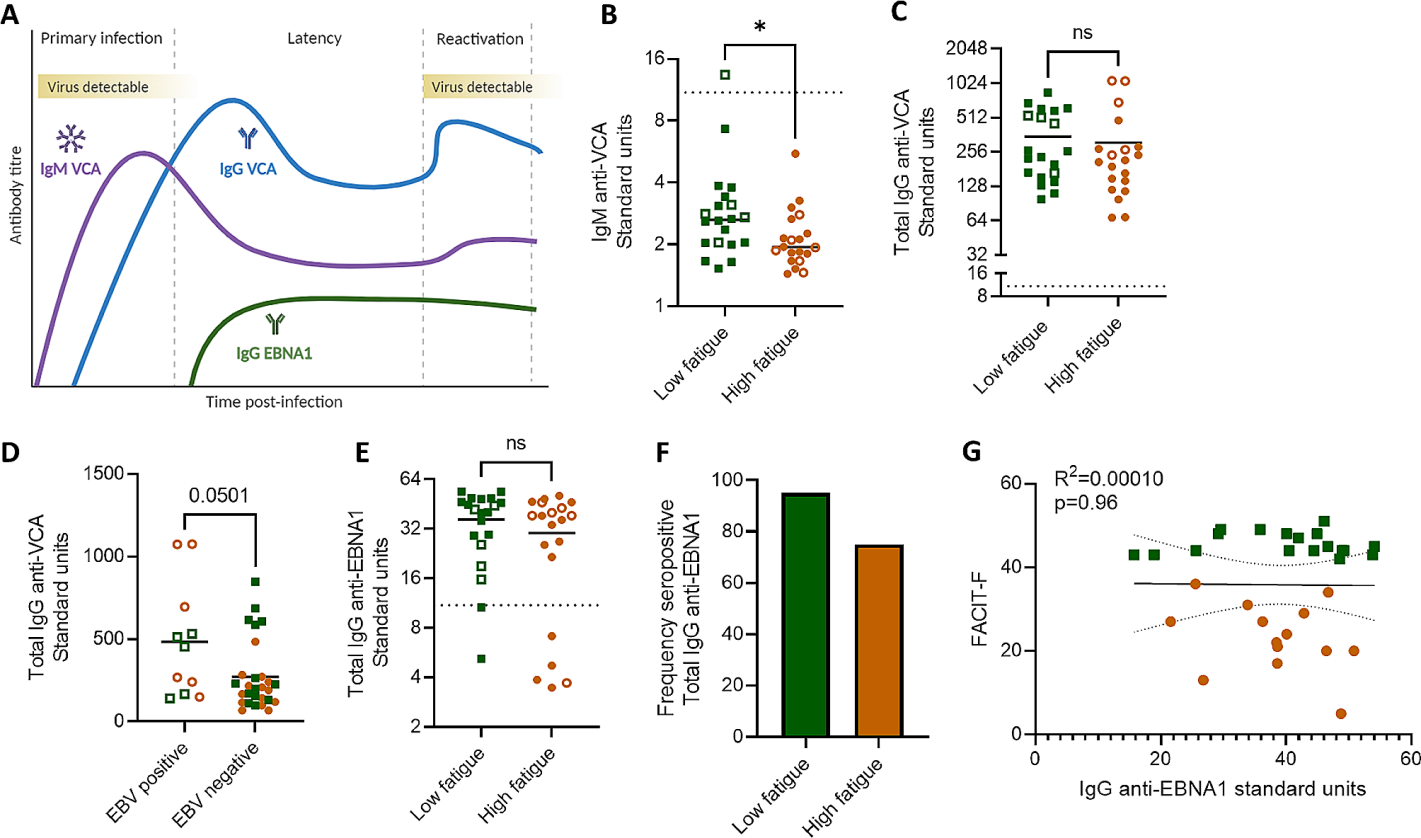
Anti-EBV seroresponse in people with post-stroke fatigue. (**A**) Schematic of expected circulating anti-EBV antibody titres following primary infection, and during latency, and reactivation. Created with BioRender. (**B**-**G**) Plasma antibodies to EBV were measured by ELISA. Low fatigue participants are plotted as green squares and high fatigue as orange circles. Open shapes indicate participants with EBV viremia, as measured by qPCR, and the dotted line (**B**, **E**, **G**) indicates the ELISA cut off control value. (**B**) Titre of anti-Epstein-Barr nuclear antigen 1 (EBNA1) IgG between participants with low versus high post-stroke fatigue. (**C**) Percent of participants within each group that are seropositive for anti-EBNA1 IgG. Seropositivity is considered any value > 10% over the cut-off control value. (**D**) Association between self-reported fatigue (FACIT-F) and IgG anti-EBNA1 titre. (**E**) Titre of IgG anti-viral capsid antigen (VCA) between low and high fatigue groups. (**F**) Titre of IgG anti-VCA between participants with and without EBV viremia, measured by qPCR. (**G**) Titre of IgM anti-viral capsid antigen (VCA) between low and high fatigue groups. Data show individual data points plus mean, (**B**, **C**, **E**, **G**) *n* = 20, (**D**) *n* = 12–30 per group.; *p < 0.05; (**E**) unpaired t-test (**B**, **F**, **G**) Mann-Whitney test; (D) linear regression, with best-fit line and 95% confidence band plotted

We first measured IgM against VCA, which would be expected to be highest during primary infection and remain persistently detectable in seropositive individuals (Fig. 3A). While nearly all samples were below the cut-off control, indicating a lack of recent primary infection, all samples were in the detectable range of the assay. We observed a lower IgM titre against VCA in the high fatigue group, compared to the low fatigue group (Fig. 3B). Next, we measured the titre of IgG against VCA, which would be expected to increase in response to viral reactivation (Fig. 3A). We did not observe a difference in anti-VCA IgG titre between groups (Fig. 3C). This result aligns with the qPCR results where patients with viremia were evenly distributed between low and high fatigue groups, and reinforce that people with higher levels of post-stroke fatigue did not show increased incidence of EBV reactivation following stroke. Individuals with viremia detected by qPCR had higher titres of anti-VCA IgG (Fig. 3D), as expected in response to an ongoing infection (Fig. 3A).

We next measured the titres of IgG against latency antigen EBNA1, indicative of memory to primary infection and/or an adaptive antibody response to reactivation, and did not observe a difference between low and high post-stroke fatigue groups (Fig. 3E). The ELISA cut off control was used to determine seropositivity, i.e. the presence of antibody responses to antigen. Seropositivity was then used to stratify participants into seropositive versus seronegative for IgG against EBNA1. We observed that of the low fatigue participants, 18/20 (90%) displayed seropositivity for anti-EBNA1 IgG, compared to 15/20 (75%) of the high fatigue participants (Fig. 3F). Of the individuals seropositive for anti-EBNA1 IgG, we did not observe a relationship between IgG anti-EBNA1 titres and fatigue (Fig. 3G). Taken together with our previous finding that individuals with high fatigue have lower IgM titres against the lytic VCA antigen (Fig. 3B), this could indicate a potentially aberrant antibody response to EBV in people with post-stroke fatigue. Titres of anti-EBV antibodies did not associate with initial stroke severity (Supplementary Fig. 1).

**Fig. 4:**
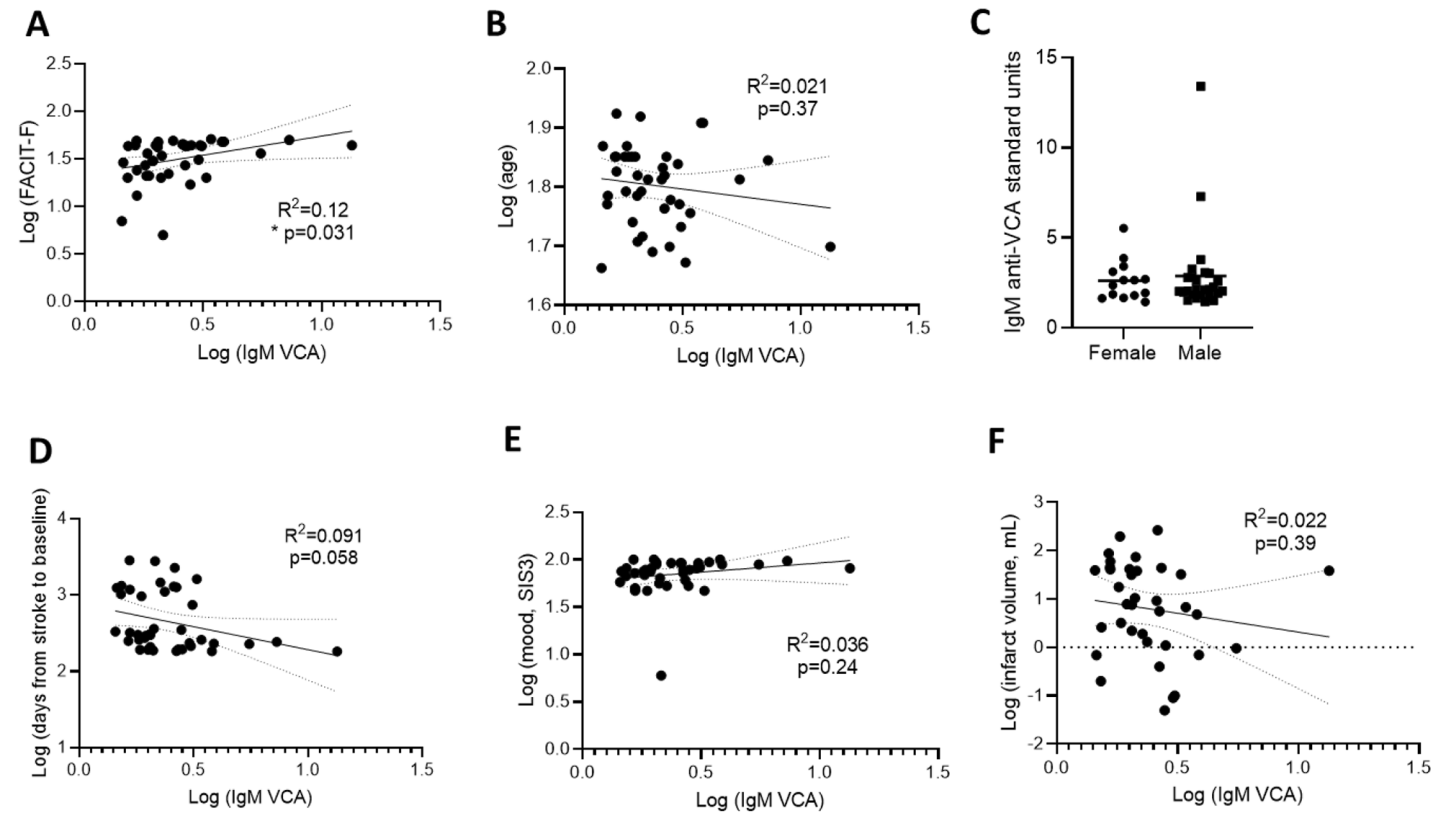
IgM anti-VCA titre correlates with fatigue but not selected clinical characteristics. Titre of IgM against VCA were plotted against various characteristics, including (**A**) FACIT-F score, (**B**) age, (**C**) biological sex, (**D**) days from stroke to baseline, (**E**) mood as measured by SIS3, and (**G**) infarct volume. Each data point represents an individual participant, (**A**, **B**, **D**, **E**, **F**) *n* = 40, (**C**) *n* = 14–26. Analysed by linear regression with best-fit line and 95% confidence band plotted (**A**-**B**, **D**-**F**)

We then examined anti-VCA IgM titres in relation to various clinical characteristics. As expected from our previous results (Fig. 3), we observed a correlation between fatigue and anti-VCA IgM titre (Fig. 4A). No association was observed between IgM against VCA and age, biological sex, days from stroke to baseline, mood, or infarct volume (Fig. 4B-F). Finally, anti-VCA IgM was not associated with cognitive domains, including working memory, processing speed, memory, visual spatial, or language (Supplementary Fig. 2).

### Total antibody titres unchanged between low and high post-stroke fatigue groups

**Fig. 5:**
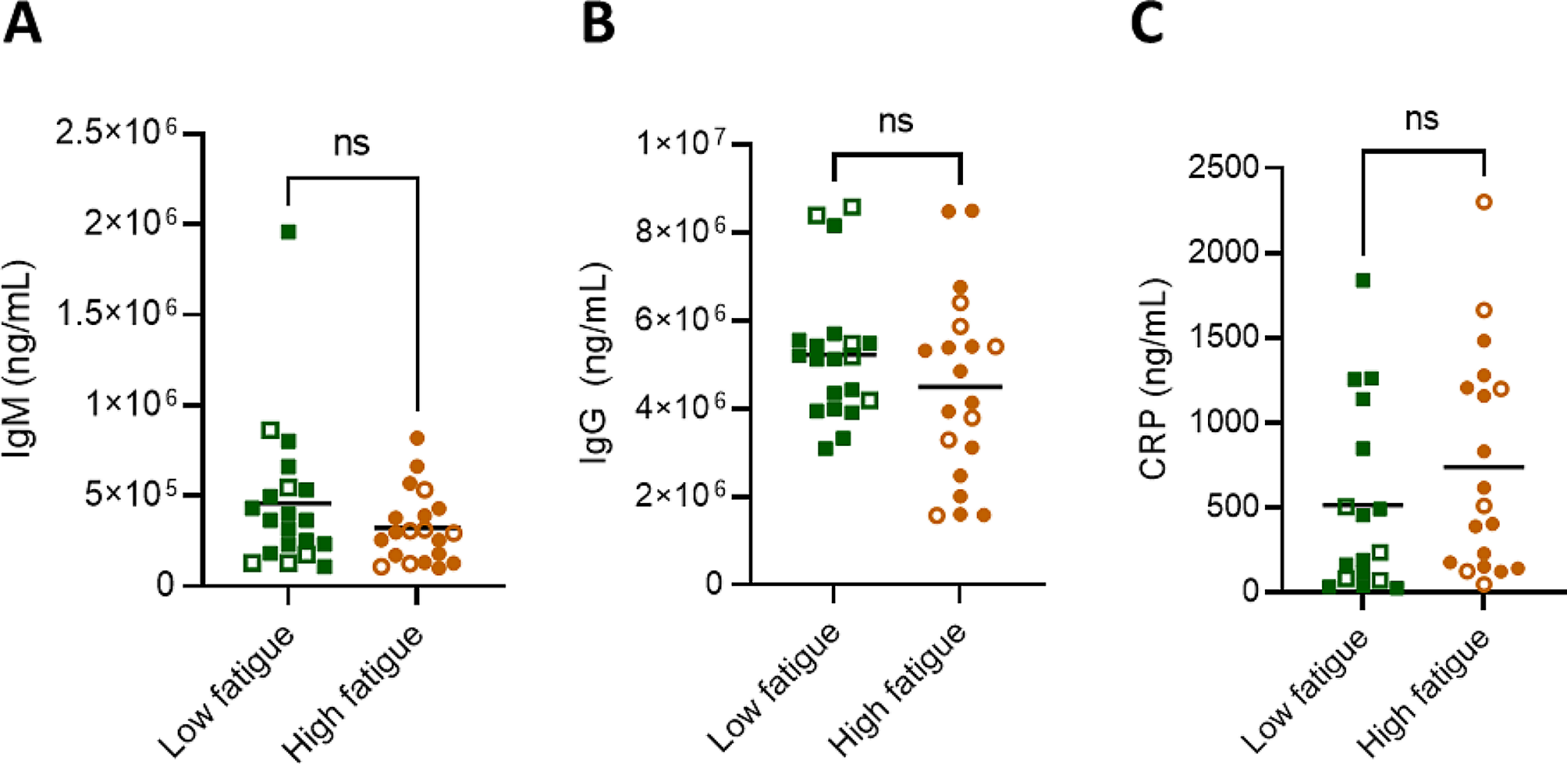
Total circulating antibodies unchanged between low and high post-stroke fatigue groups. Amount of circulating IgM and total IgG antibodies, as well as C-reactive protein (CRP). Data show individual participants plus mean, *n* = 20 per group; analysed by Mann-Whitney test

To investigate if changes to total circulating antibody levels were responsible for the observed changes in anti-EBV antibodies, we measured circulating IgM and total IgG. We did not observe differences in the levels of IgM nor total IgG between the low and high fatigue groups (Fig. 5A, B), indicating that the observed differences in anti-VCA IgM were not driven by a loss of total circulating IgM.

Additionally, we measured the inflammatory protein C-reactive protein (CRP) that has been previously associated with poor outcome after stroke – and is indicative of stroke-induced systemic immune alterations [29]. We did not observe a difference in the level of circulating CRP between those with low and high post-stroke fatigue, suggesting that fatigue is not associated with general systemic immune alterations post-stroke (Fig. 5C).

No differences in antibody or CRP levels were observed in the participants with EBV viremia (open shapes) compared to those without detectable circulating virus (filled shapes, Fig. 5A-C).

## Discussion

In this study, we aimed to explore if EBV reactivation associates with post-stroke fatigue. To our knowledge, this is the first study to examine EBV, and its relationship with fatigue, during stroke recovery. Here, we report a lack of evidence for EBV reactivation in people with post-stroke fatigue during chronic stroke recovery. However, these results suggest possible alterations to the EBV antibody response, particularly the anti-VCA IgM response, in people with post-stroke fatigue. Our exploratory analyses found the dampened IgM against VCA observed in participants with post-stroke fatigue does not associate with other clinical or neurological parameters, suggesting a link to fatigue itself. There were no differences in total IgM or IgG antibody concentrations between high and low fatigue groups, suggesting this is specifically associated with the antibody response to EBV.

Estimates of EBV reactivation in the general population vary but have been reported between 30 and 50% [30–32], and reactivation is known to increase with age [33]. In this study, we detected EBV in 25% of participants, indicating that people with a history of stroke may not have elevated EBV reactivation, although a direct comparison with healthy participants would be necessary to confirm. Increased IgG titres against EBV lytic antigen were shown to be a common feature in patients who experienced long-COVID, associated with high fatigue [6] and a smaller study confirmed reactivation of EBV in 50% of patients with long-COVID versus 20% with healthy recovery [34]. However, EBV reactivation was detected in 82% of patients admitted to the intensive care unit with ongoing severe COVID-19, suggesting peak viral reactivation occurs during acute illness [35]. Further studies have confirmed that viral reactivation occurs during acute COVID-19 infection and is a predictor of long-COVID [36]. Together, these studies suggest that sampling time is an important consideration when investigating EBV reactivation, but the immunological effects of this may persist beyond the reactivation event itself.

We examined EBV reactivation by measuring circulating virus and anti-EBV antibody titres and by both measures there was no evidence of EBV reactivation. Surprisingly, there were indications of an aberrant anti-EBV immune response in participants with high fatigue. The IgM titre against lytic EBV antigen, VCA, was decreased in participants with higher post-stroke fatigue and also negatively correlated with post-stroke fatigue. Additionally, a lower proportion of high fatigue participants were seropositive for the IgG titre against latency antigen EBNA1, however we did not observe a correlation with fatigue. The mechanism and clinical relevance of this finding is not clear from current data. Stroke induces a systemic suppression of immune response, the extent of which is associated with the initial stroke severity [37]. We have previously shown that B cell numbers, and antibody responses, are reduced after moderate and severe strokes [19, 38]. However, we saw no association between titres of anti-VCA IgM and infarct volume or stroke severity. Furthermore, there were no differences in total IgM or in CRP concentrations between low and high fatigue groups. This suggests that the altered antibody titres to EBV antigens is not simply a result of more extensive systemic immune alterations in the high fatigue patient group. We chose to examine patients at least five months (and up to eight years) post-stroke as fatigue is a chronic condition, and to ask at that time whether active EBV infection may drive fatigue. However, immune alterations that occur acutely after stroke are associated with increased infection risk. Therefore, it is possible that EBV reactivation could occur more acutely after stroke. The reduced antibody titres to EBV antigen in chronic recovery in high fatigue patients may be an indication of an aberrant immune response to earlier viral reactivation.

It is possible that there are common factors that both pre-dispose people to experience fatigue after stroke and can also drive reactivation of EBV. For example advanced age and cognitive factors such as stress and depression have been suggested to be important contributing factors to both of these phenomena [39–41]. Furthermore, it is possible that post-stroke immunosuppression could be a factor that independently contributes to EBV reactivation as well as post-stroke fatigue. These potential confounders can make causal links difficult to establish.

There are further limitations to consider in this study including the relatively small sample size. Additionally, fatigue is a subjective measure, reliant on patient reporting. In this study fatigue was examined by the FACIT-F tool, however various tools exist to measure fatigue and there can be discrepancies in the extent of fatigue recorded depending on which test is used [42]. We did not have a record of chronic infection incidence for patients in this study, which could indicate patient vulnerability to infection in general and influence the reactivation of EBV. Here we examined only EBV, though there is evidence that other viruses reactivate following stroke, such as herpes zoster virus [21]. Reactivation of multiple viruses, including cytomegalovirus and human herpesvirus (HHV) 6, HHV-7, HHV-8 have been associated with chronic fatigue and could play a role in fatigue after stroke [43].

Although we have not found an association with EBV reactivation during chronic stroke recovery and incidence of fatigue, the alterations to circulating EBV antibody titres suggest we should not fully dismiss a potential role for reactivation of EBV and/or other latent viruses in the pathophysiology of post-stroke fatigue. Further studies using acute recovery timepoints, larger participant numbers, and alternative latent viruses will be required to fully examine the role of viral reactivation in post-stroke fatigue.

### Electronic supplementary material

Below is the link to the electronic supplementary material.

Supplementary Material 1

Supplementary Material 2

## Data Availability

The raw and anonymized data used in this study can be made available to other researchers on request. Written proposals can be addressed to the corresponding author and will be assessed by the StrokeCog investigators for appropriateness of use, and a data sharing agreement will be put in place before data are shared.

## Declarations

### Open access

For the purpose of open access, the author has applied a CC BY public copyright licence to any Author Accepted Manuscript version arising from this submission.
