# Supplementary material for: Evidence of aberrant anti-epstein-barr virus antibody response, though no viral reactivation, in people with post-stroke fatigue
Source: J Inflamm (Lond). 2024 Aug 12;21:30. doi: 10.1186/s12950-024-00402-0 (PMC11321160; doi:10.1186/s12950-024-00402-0)
Supplement: Supplementary file 1 — Supplementary Material 1 [file 12950_2024_402_MOESM1_ESM.docx]

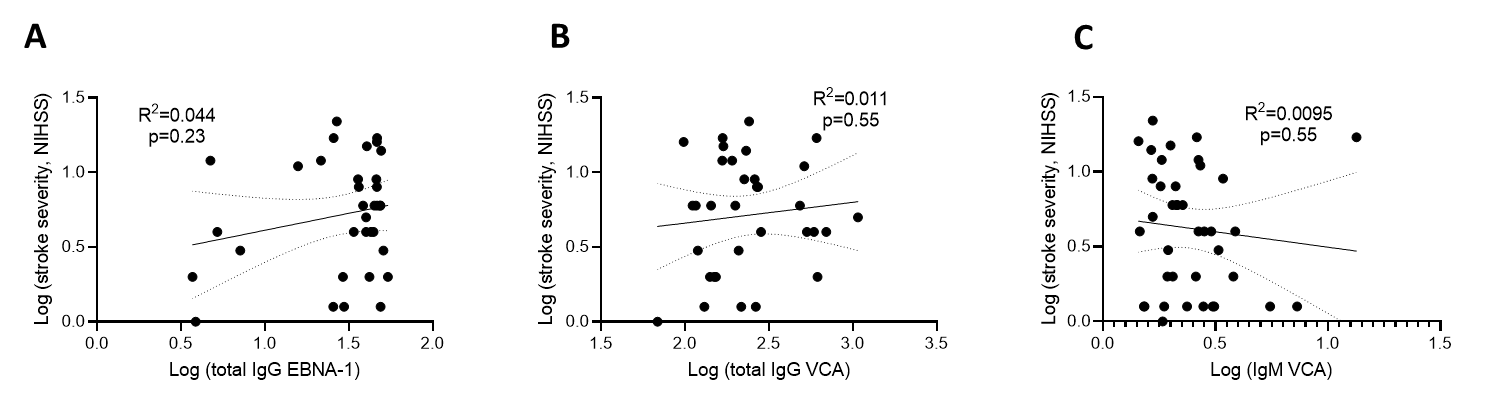


***Supplementary Figure 1. Anti-EBV antibodies do not associate with stroke severity.***

Stroke severity, measured by NIHSS, in relation to EBV antibody titres, (A) total IgG against EBNA1, (B) total IgG against VCA, and (C) IgM against VCA. Each data point represents an individual participant. Analysed by linear regression with best-fit line and 95% confidence band plotted.
