# Supplementary material for: Evidence of aberrant anti-epstein-barr virus antibody response, though no viral reactivation, in people with post-stroke fatigue
Source: J Inflamm (Lond). 2024 Aug 12;21:30. doi: 10.1186/s12950-024-00402-0 (PMC11321160; doi:10.1186/s12950-024-00402-0)
Supplement: Supplementary file 2 — Supplementary Material 2 [file 12950_2024_402_MOESM2_ESM.docx]

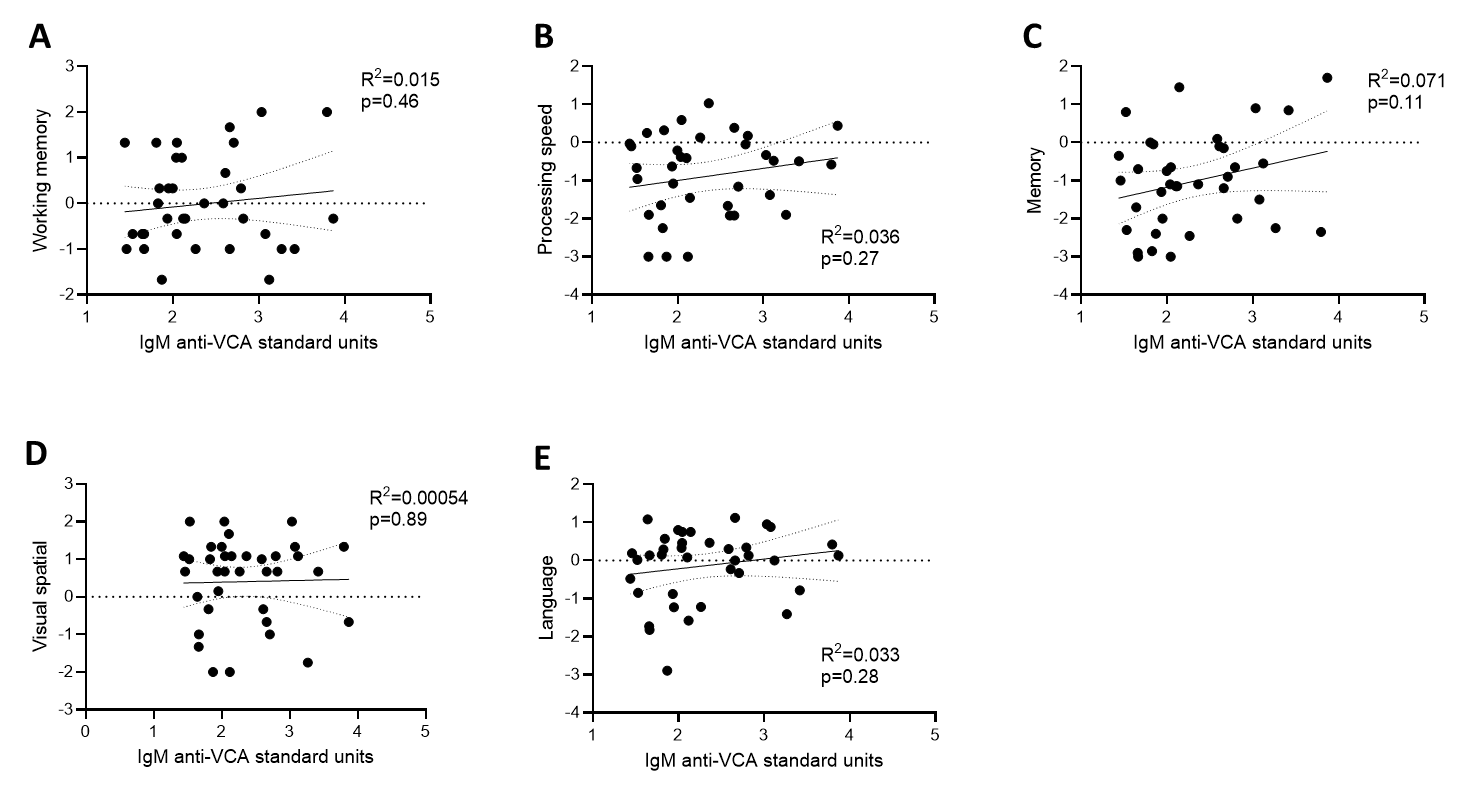


***Supplementary Figure 2. IgM anti-VCA titre does not associate with various cognitive domains***

Titre of IgM against VCA in relation to various cognitive domains, (A) working memory, (B) processing speed, (C) memory, (D) visual spatial, (E) language. Z-score. Cognitive domains are shown as z-scores, or the number of standard deviations above or below the mean. Each data point represents an individual participant. Analysed by linear regression with best-fit line and 95% confidence band plotted.
